# The impact of kidney function on Alzheimer’s disease blood biomarkers: implications for predicting amyloid-β positivity

**DOI:** 10.1186/s13195-025-01692-z

**Published:** 2025-02-19

**Authors:** Burak Arslan, Wagner S. Brum, Ilaria Pola, Joseph Therriault, Nesrine Rahmouni, Jenna Stevenson, Stijn Servaes, Kübra Tan, Paolo Vitali, Maxime Montembeault, Jesse Klostranec, Arthur C. Macedo, Cecile Tissot, Serge Gauthier, Juan Lantero-Rodriguez, Eduardo R. Zimmer, Kaj Blennow, Henrik Zetterberg, Pedro Rosa-Neto, Andrea L. Benedet, Nicholas J. Ashton

**Affiliations:** 1 Department of Psychiatry and Neurochemistry, Institute of Neuroscience & Physiology, the Sahlgrenska Academy at the University of Gothenburg, Mölndal, Sweden; 2 Graduate Program in Biological Sciences: Biochemistry, Universidade Federal Do Rio Grande Do Sul (UFRGS), Porto Alegre, Brazil; 3 Translational Neuroimaging Laboratory, Department of Neurology and Neurosurgery, Psychiatry and Pharmacology and Therapeutics, McGill University Research Centre for Studies in Aging, Montreal Neurological Institute-Hospital, Douglas Research Institute, McGill University, Montreal, Canada; 4 Lawrence Berkeley National Laboratory, Berkeley, CA USA; 5 Graduate Program in Biological Sciences: Pharmacology and Therapeutics, UFRGS, Porto Alegre, Brazil; 6 Department of Pharmacology, UFRGS, Porto Alegre, Brazil; 7 McGill Centre for Studies in Aging, McGill University, Montreal, QC Canada; 8 Paris Brain Institute, ICM, Pitié-Salpêtrière Hospital, Sorbonne University, Paris, France; 9 Division of Life Sciences and Medicine, and Department of Neurology, Institute On Aging and Brain Disorders, Neurodegenerative Disorder Research Center, University of Science and Technology of China and First Affiliated Hospital of USTC, Hefei, People’s Republic of China; 10 Institute of Psychiatry, Psychology and Neuroscience Maurice Wohl Institute Clinical, King’s College London, Neuroscience Institute London, London, UK; 11 Banner Alzheimer’s Institute and University of Arizona, Phoenix, AZ USA; 12 Banner Sun Health Research Institute, Sun City, AZ 85351 USA; 13 School of Medicine and Public Health, Wisconsin Alzheimer’s Institute, University of Wisconsin, Madison, WI USA; 14 Clinical Neurochemistry Laboratory, Sahlgrenska University Hospital, Gothenburg, Sweden; 15 Department of Neurodegenerative Disease, Institute of Neurology, University College London, London, UK; 16 UK Dementia Research Institute, University College London, London, UK; 17 Hong Kong Center for Neurodegenerative Diseases, Hong Kong, China

**Keywords:** eGFR, Kidney impairment, Amyloid, p-tau, NfL, GFAP

## Abstract

**Background:**

Impaired kidney function has a potential confounding effect on blood biomarker levels, including biomarkers for Alzheimer’s disease (AD). Given the imminent use of certain blood biomarkers in the routine diagnostic work-up of patients with suspected AD, knowledge on the potential impact of comorbidities on the utility of blood biomarkers is important. We aimed to evaluate the association between kidney function, assessed through estimated glomerular filtration rate (eGFR) calculated from plasma creatinine and AD blood biomarkers, as well as their influence over predicting Aβ-positivity.

**Methods:**

We included 242 participants from the Translational Biomarkers in Aging and Dementia (TRIAD) cohort, comprising cognitively unimpaired individuals (CU; *n* = 124), mild cognitive impairment (MCI; *n* = 58), AD dementia (*n* = 34), and non-AD dementia (*n* = 26) patients all characterized by [^18^F] AZD-4694. Plasma samples were analyzed for Aβ42, Aβ40, glial fibrillary acidic protein (GFAP), neurofilament light chain (NfL), tau phosphorylated at threonine 181 (p-tau181), 217 (*p*-tau217), 231 (p-tau231) and N-terminal containing tau fragments (NTA-tau) using Simoa technology. Kidney function was assessed by eGFR in mL/min/1.73 m^2^, based on plasma creatinine levels, age, and sex. Participants were also stratified according to their eGFR-indexed stages of chronic kidney disease (CKD). We evaluated the association between eGFR and blood biomarker levels with linear models and assessed whether eGFR provided added predictive value to determine Aβ-positivity with logistic regression models.

**Results:**

Biomarker concentrations were highest in individuals with CKD stage 3, followed by stages 2 and 1, but differences were only significant for NfL, Aβ42, and Aβ40 (not Aβ42/Aβ40). All investigated biomarkers showed significant associations with eGFR except plasma NTA-tau, with stronger relationships observed for Aβ40 and NfL. However, after adjusting for either age, sex or Aβ-PET SUVr, the association with eGFR was no longer significant for all biomarkers except Aβ40, Aβ42, NfL, and GFAP. When evaluating whether accounting for kidney function could lead to improved prediction of Aβ-positivity, we observed no improvements in model fit (Akaike Information Criterion, AIC) or in discriminative performance (AUC) by adding eGFR to a base model including each plasma biomarker, age, and sex. While covariates like age and sex improved model fit, eGFR contributed minimally, and there were no significant differences in clinical discrimination based on AUC values.

**Conclusions:**

We found that kidney function seems to be associated with AD blood biomarker concentrations. However, these associations did not remain significant after adjusting for age and sex, except for Aβ40, Aβ42, NfL, and GFAP. While covariates such as age and sex improved prediction of Aβ-positivity, including eGFR in the models did not lead to improved prediction for any biomarker. Our findings indicate that renal function, within the normal to mild impairment range, does not seem to have a clinically relevant impact when using highly accurate blood biomarkers, such as p-tau217, in a biomarker-supported diagnosis.

**Supplementary Information:**

The online version contains supplementary material available at 10.1186/s13195-025-01692-z.

## Introduction

The quest to identify reliable biomarkers for early detection of Alzheimer’s disease (AD) and monitoring disease progression has gained significant momentum in recent years [1]. Established AD biomarkers included positron emission tomography (PET) [2–4] and cerebrospinal fluid (CSF) measurements [2, 5, 6], which reflect the neuropathological hallmarks of AD. Blood-based biomarkers that are widely validated against CSF and imaging measures hold great promise for enabling early intervention and improved patient management [7]. Certain amyloid-beta (Aβ) plasma assays demonstrate good agreement with Aβ-PET, but their small reduction in Aβ-positive compared to Aβ-negative patients, peripheral expression and confounding by medication use may limit their utility [8, 9]. Phosphorylated tau (p-tau) biomarkers, detectable through various assays [10] and epitopes, such as p-tau181 [11], p-tau217 [12], and p-tau231 [13], are increased in Aβ-positive patients and their levels increase even further with advanced clinical stages and evident tau pathology [14]. Unlike p-tau, N-terminal containing tau fragments (NTA-tau) [15, 16] measures soluble tau fragments regardless of their phosphorylation state, which increase during mid-to-late AD and are tightly associated with tau pathology. In contrast, plasma neurofilament light (NfL) acts as a general marker of neuro-axonal degeneration, which occurs across several neurodegenerative diseases [17], whereas plasma glial fibrillary acidic protein (GFAP) is elevated early in the course of AD, showing an association with Aβ pathology [18] but also increased on other neurogenerative disorders.

In anticipation of forthcoming disease-modifying therapies (DMT) across the globe, such as lecanemab [19] and donanemab [20], and prior to the integration of blood-based biomarkers into clinical practice, it is imperative to delineate the confounding factors that may exert a substantial influence on blood biomarker measurements [21]. As the field of AD biomarker research progresses, it becomes increasingly important to account for such factors that could impact the accuracy and interpretation of fluid biomarkers, particularly p-tau217, which is anticipated to be the most useful in this context. One such confounding factor that has garnered attention is impaired kidney function [22]. The kidneys play a pivotal role in the clearance of waste products and various substances from the bloodstream, including promising AD biomarkers. Impaired kidney function, as reflected by low estimated glomerular filtration rate (eGFR), can disrupt the equilibrium of substances in the bloodstream, potentially affecting the concentrations of analytes measured in peripheral tissues such as blood. For proteins such as β2-microglobulin (B2M) [23] and immunoglobulin light chains (free light chains) [24], which are routinely measured in clinical chemistry laboratories, their levels can change due to impaired kidney function and decreased clearance. Given this, it is relevant to investigate the extent to which kidney impairment influences the potential clinical utility of AD blood biomarkers.

It has been previously shown that various medical comorbidities, including chronic kidney disease (CKD), are associated with plasma levels of AD biomarkers, such as Aβ, p-tau species and NfL [22, 25, 26]. However, many studies have lacked data on eGFR [22, 26, 27], a standardized measure of kidney function [28], and most have focused on only a narrow selection of plasma AD biomarkers [29–31]. Studying the relationship between eGFR and AD biomarker concentrations, along with evaluating the potential enhancement of amyloid prediction models through the incorporation of eGFR, is essential. This could provide valuable insights on whether kidney function has clinically relevant implications for the incorporation of these biomarkers in the routine diagnostic work-up of AD and for the inclusion of individuals with preclinical AD in clinical trials.

In this study, we aimed to investigate the impact of kidney impairment on a broad range of AD plasma biomarkers, including Aβ40, Aβ42, GFAP, NfL, p-tau181, p-tau217, p-tau231, and NTA-tau. We first began by examining the concentrations of these biomarkers across different stages of CKD. Next, we incorporated eGFR into multiple regression models to examine how these biomarkers associate with eGFR. Finally, we assessed whether including eGFR in clinical prediction models to determine Aβ-positivity would lead to enhanced model fit or higher discriminative ability.

## Methods

### Participants & ethics

This research involved participants from the Translational Biomarkers in Aging and Dementia (TRIAD) observational cohort, which was designed to represent the AD continuum. Clinical diagnoses were conducted with no prior knowledge of the biomarker results. All subjects underwent clinical evaluations, which included the Clinical Dementia Rating (CDR) [32], Mini-Mental State Examination (MMSE) [33], and an assessment of cerebrovascular disease risk using the Hachinski Ischemic Scale [34]. Participants were excluded from the study if they had uncontrolled systemic conditions despite being on a stable medication regimen. Additional exclusion criteria included ongoing substance abuse, recent head trauma, recent major surgery, or contraindications for magnetic resonance imaging (MRI)/PET safety. All participants provided informed consent, and the study protocols were approved by the appropriate ethical review boards. TRIAD was approved by the Montreal Neurological Institute PET working committee and the Douglas Mental Health University Institute Research Ethics Board (IUSMD16-61, IUSMD16-60). A detailed description of the cohort can be found in the supplementary material.

### Imaging

Within the TRIAD cohort, Aβ-PET scans were conducted using [18F]-AZD4694. Amyloid positivity (referred to as A +) was determined when the [18F]-AZD-4694 standardized uptake value ratio (SUVR) exceeded 1.55. For participants in TRIAD, the first blood sample is usually collected at the screening visit, with PET scans being scheduled for a date following this visit, leading to a mean interval between the initial plasma sample collection and the PET scan of + 0.44 years for Aβ-PET. Detailed imaging methods can be found in the supplementary methods.

### Measurement of plasma creatinine

Blood samples were collected according to protocols that have been previously established and described [11]. Plasma creatinine levels were determined using the Roche Cobas chemistry analyzer (Roche Diagnostics in Indianapolis, IN) at the Department of Psychiatry and Neurochemistry, University of Gothenburg. The analysis was conducted through the enzymatic assay known as creatinine PlusVer.2 (REF 03263991), which is designed by Roche and operated on the c501 platform. Detailed method can be found in the Supplementary Methods.

### Calculation of estimated Glomerular Filtration Rate

Glomerular barrier function was evaluated by eGFR (mL/min/1.73 m^2^) using the plasma creatinine, age, and gender [28]. CKD has been categorized into five stages in accordance with the 2012 Guidelines set forth by the Kidney Disease: Improving Global Outcomes (KDIGO-CKD) organization [35]: CKD-stage 1, characterized by eGFR ≥ 90 mL/min/1.73 m^2^; CKD-stage 2, with eGFR ranging from 60–89 mL/min/1.73 m^2^; CKD-stage 3, encompassing eGFR levels of 30–59 mL/min/1.73 m^2^; CKD-stage 4, involving eGFR values within the range of 15–29 mL/min/1.73 m^2^; and CKD-stage 5, defined by eGFR < 15 mL/min/1.73 m^2^. Due to the limited number of patients in CKD stages 4 and 5, participants in this cohort were grouped into three categories based on their eGFR: CKD Stage 1 (eGFR ≥ 90; n = 147, 61.00%), CKD Stage 2 (eGFR 60–89; n = 88, 36.51%), and CKD Stage 3 or higher (eGFR < 60; n = 6, 2.49%) (Table 2). To highlight that most participants in this cohort fall within the normal (eGFR > 90) to mild renal impairment range (eGFR 60–90), the patient cohort was divided into two groups accordingly. Additionally, participants with eGFR < 60 were included in the mild renal impairment group for the purposes of visualizing the distribution, as shown in Supplementary Fig. 2.

### Plasma Biomarkers

Plasma samples were analyzed at the Department of Psychiatry and Neurochemistry, University of Gothenburg. The quantification of plasma Aβ 42/40, GFAP, and NfL was carried out using the commercially available Neurology 4-plex E kit (#103670, Quanterix).Plasma p-tau181 [11], p-tau231 [13], and NTA-tau [15] were analyzed using in-house Simoa assays developed at the University of Gothenburg. Plasma p-tau217 was quantified using the commercially available AlzPath Simoa assay [12], as previously described. All measurements were performed on the automated Simoa HD-X platform (Quanterix, MA, USA).

### Statistical analysis

Demographic information was summarized with median (Q1-Q3) for continuous variables and as count and percentages for categorical variables. We initially plotted plasma biomarker concentrations according to CKD stage and examined between-group differences with linear models adjusted for age and sex. Given the small number of individuals in CKD stage 3, we focused further analyses on continuous eGFR measures. We computed the Spearman correlation between plasma biomarkers and eGFR, and plotted a regression line derived based on a generalized additive model with cubic splines (3 knots) over the scatterplot to visually represent the associations. Then, we used linear models with blood biomarkers as the outcome variable to evaluate the magnitude of the association between eGFR and plasma biomarkers, plotting the standardized eGFR β-estimate and 95% confidence intervals with three models per plasma biomarker. The first model included only eGFR as a predictor; the second included eGFR, age, and sex; the third, eGFR, age, sex, and Aβ-PET SUVr. This was to determine whether eGFR would remain associated with blood biomarkers independently of demographics and AD pathology. Afterwards, we evaluated whether eGFR would contribute to the prediction of Aβ-PET positivity. We built logistic regression models with Aβ-PET status as the outcome and three schemes of predictors: (i) plasma biomarker alone, (ii) plasma biomarker, age and sex, (iii) plasma biomarker, age, sex, and eGFR. Within each biomarker, we then compared the area under the curve (AUC) between the models and calculated the delta Akaike Information Criterion (AIC) from the models compared to the plasma biomarker alone. A difference of -2 or greater would be considered significant based on previous work [36]. We also visually compared the functional form of biomarkers and eGFR in each full model, based on model-derived probabilities, to visualize the non-linear relationship between these predictors and the risk of Aβ-PET positivity. These logistic and linear models were always fitted separately for each biomarker, given our goal was to evaluate the influence of eGFR on each biomarker rather than developing a combination panel of biomarkers. Statistical significance was defined as an alpha of 0.05, and analyses were all performed in R Statistical Software (version 4.2.1).

## Results

### Participant characteristics

The study encompassed 242 participants, with the following median ages across groups: CKD-stage 1 (median = 67.1 years, IQR: 62.5–72.1), CKD-stage 2 (median = 73.0 years, IQR: 69.0–77.3), and CKD-stage 3 (median = 78.2 years, IQR: 74.9–79.2). There were 160 females (66.4%) in the total cohort. 147 (61%), 88 (36.5%), and 6 (2.5%) participants were assigned to CKD-stage 1, stage 2, and stage 3 groups, respectively. eGFR levels were highest in the CKD-stage 1 group, while participants in the CKD-stage 3 group were the oldest and exhibited higher levels of plasma biomarkers. The detailed demographic, clinical information, and plasma biomarker results of participants are described in Table 1 and Table 2. There were no significant differences in eGFR levels between males and females, and age was significantly associated with eGFR (Supplementary Fig. 1).

**Table 1:** Demographic and clinical characteristics of study participants

	**CU (*N* = 124)**	**MCI (*N* = 58)**	**AD (*N* = 34)**	**Non-AD (*N* = 26)**
Age, median (Q1-Q3)	70.3 (65.7–74.7)	72.2 (66.3–75.9)	66.9 (61.1–73.5)	64.0 (57.5–69.6)
Female, n (%)	89 (71.8%)	35 (60.3%)	21 (61.8%)	15 (57.7%)
YOE, median (Q1-Q3)	15.0 (12.8–18.0)	16.0 (12.0–17.8)	15.0 (13.3–16.0)	15.0 (12.0–17.0)
MMSE, Median (Q1-Q3)	29.0 (28.0–30.0) [11]	29.0 (27.0–29.0) [8]	23.0 (20.0–25.8) [4]	26.0 (23.8–29.0) [6]
APOE ε4 carriers, No. (%)	29 (23.4%) [0]	26 (44.8%) [2]	20 (58.8%) [0]	3 (11.5%) [1]
Amyloid status, No. (%)	30 (24.2%) [0]	37 (63.8%) [0]	33 (97.1%) [0]	0 (0%) [0]
eGFR Median (Q1-Q3)	92.8 (84.2–97.3)	93.1 (83.1–97.2)	92.3 (87.8–97.5)	100 (88.6–104)
Plasma p-tau217, median (Q1-Q3), pg/mL	0.274 (0.170–0.405) [13]	0.677 (0.428–0.940) [7]	1.31 (0.671–1.83) [3]	0.273 (0.190–0.353) [0]
Plasma p-tau181, median (Q1-Q3), pg/mL	7.12 (5.46–10.7) [1]	8.71 (6.82–11.8) [1]	13.6 (9.21–16.9) [0]	7.49 (4.74–10.6) [0]
Plasma p-tau231, median (Q1-Q3), pg/mL	13.0 (10.0–16.7) [0]	18.5 (13.8–23.7) [2]	22.8 (15.9–28.6) [1]	13.4 (11.1–19.8) [0]
Plasma NTA-tau, median (Q1-Q3), pg/mL	0.177 (0.111–0.308) [5]	0.242 (0.112–0.432) [1]	0.522 (0.384–0.635) [1]	0.195 (0.148–0.473) [0]
Plasma GFAP, median (Q1-Q3), pg/mL	154 (109–195) [1]	186 (134–232) [0]	270 (190–312) [0]	140 (72.7–189) [0]
Plasma NfL, median (Q1-Q3), pg/mL	19.4 (14.5–26.9) [1]	23.6 (16.7–30.1) [0]	31.2 (22.0–36.2) [0]	23.3 (14.4–38.4) [0]
Plasma Aβ_42_, median (Q1-Q3), pg/mL	6.65 (5.68–7.83) [1]	6.56 (5.52–7.19) [0]	5.46 (4.32–6.38) [0]	6.84 (5.23–8.33) [0]
Plasma Aβ_40_, median (Q1-Q3), pg/mL	92.0 (80.5–101) [1]	91.6 (85.7–106) [0]	97.0 (78.9–103) [0]	86.7 (75.5–103) [0]
Plasma Aβ_42/40_, median (Q1-Q3), pg/mL	0.0725 (0.0630–0.0828) [1]	0.0664 (0.0616–0.0778) [0]	0.0601 (0.0551–0.0658) [0]	0.0767 (0.0685–0.0854) [0]

Categorical variables are reported as counts and percentages, while continuous variables are presented as median values with interquartile ranges (Q1-Q3). Missing values are reported in brackets next to the respective variables

*Abbreviations CU* Cognitively Unimpaired, *MCI* Mild Cognitive Impairment, *AD* Alzheimer's Disease, *Non-AD* Non-Alzheimer's Disease, *YOE* Years of Education, *MMSE* Mini-Mental State Examination, *APOE* Apolipoprotein E, *eGFR* Estimated Glomerular Filtration Rate, *p-tau* phosphorylated tau, *NTA-tau* N-terminal containing tau fragments, *GFAP* Glial Fibrillary Acidic Protein, *NfL* Neurofilament Light chain, *Aβ* Amyloid Beta

**Table 2:** Participant characteristics and plasma biomarker levels across ckd stages

	**CKD Stage-1 (*N* = 147)**	**CKD Stage-2 (*N* = 88)**	**CKD Stage-3 (*N* = 6)**
Age, median (Q1-Q3)	67.1 (62.5–72.1)	73.0 (69.0–77.3)	78.2 (74.9–79.2)
Female, n(%)	97 (66.0%)	61 (69.3%)	2 (33.3%)
YOE, median (Q1-Q3)	15.0 (12.0–17.0)	15.0 (12.0–18.0)	18.0 (17.3–19.5)
MMSE, Median (Q1-Q3)	29.0 (27.3–30.0) [21]	28.5 (26.0–30.0) [8]	29.0 (28.3–29.8) [0]
APOE ε4 carriers, No. (%)	50 (34.0%) [3]	26 (29.5%) [0]	2 (33.3%) [0]
Amyloid status, No. (%)	61 (41.5%)	37 (42.0%)	2 (33.3%)
Plasma p-tau217, median (Q1-Q3), pg/mL	0.305 (0.188–0.693) [10]	0.418 (0.263–0.812) [13]	0.572 (0.550–0.890) [0]
Plasma p-tau181, median (Q1-Q3), pg/mL	7.98 (5.62–11.9) [1]	8.46 (6.73–12.5) [1]	11.6 (10.5–15.5) [0]
Plasma p-tau231, median (Q1-Q3), pg/mL	14.1 (10.6–20.2) [2]	16.5 (12.5–20.6) [1]	23.8 (19.7–31.5) [0]
Plasma NTA-tau, median (Q1-Q3), pg/mL	0.213 (0.118–0.410) [4]	0.251 (0.123–0.399) [3]	0.282 (0.220–0.447) [0]
Plasma GFAP, median (Q1-Q3), pg/mL	149 (104–218) [0]	181 (143–252) [1]	236 (190–257) [0]
Plasma NfL, median (Q1-Q3), pg/mL	18.8 (14.4–27.1) [0]	26.8 (18.5–32.7) [1]	40.4 (31.7–48.7) [0]
Plasma Aβ42, median (Q1-Q3), pg/mL	6.17 (4.86–7.15) [0]	6.84 (5.79–7.77) [1]	8.50 (6.76–10.3) [0]
Plasma Aβ40, median (Q1-Q3), pg/mL	89.6 (77.2–100) [0]	93.8 (84.8–105) [1]	124 (113–143) [0]
Plasma Aβ42/40, median (Q1-Q3), pg/mL	0.0682 (0.0608–0.0789) [0]	0.0732 (0.0611–0.0828) [1]	0.0679 (0.0636–0.0811) [0]

Categorical variables are reported as counts and percentages, while continuous variables are presented as median values with interquartile ranges (Q1-Q3). Missing values are reported in brackets next to the respective variables

Abbreviations: *CKD* Chronic kidney disease, *YOE* Years of Education, *MMSE* Mini-Mental State Examination, *APOE* Apolipoprotein E, *eGFR* Estimated Glomerular Filtration Rate, *p-tau* phosphorylated tau, *NTA-tau* N-terminal containing tau fragments, *GFAP* Glial Fibrillary Acidic Protein, *NfL* Neurofilament Light chain, *Aβ* Amyloid Beta

### Plasma biomarker concentrations after stratified by eGFR

**Fig. 1:**
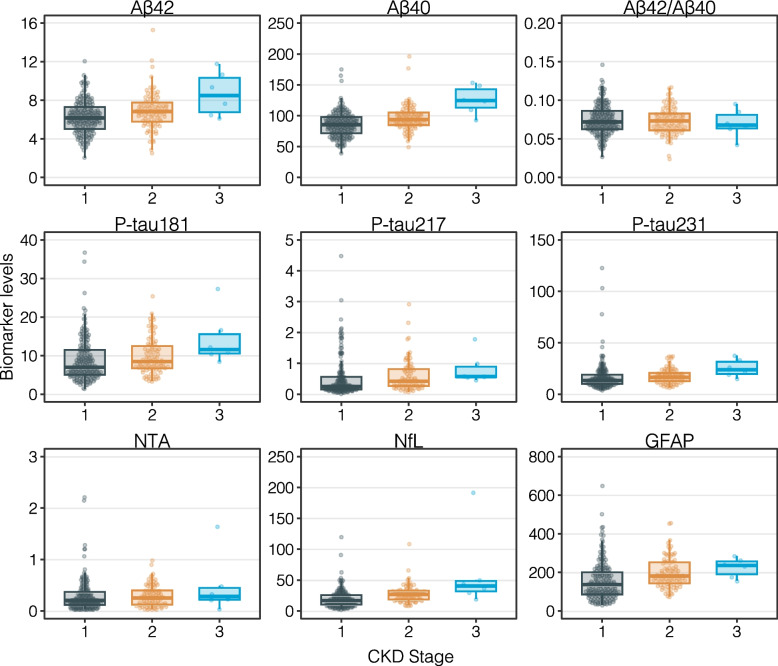
Distribution of Alzheimer's Disease Biomarkers Across CKD Stages. The box-and-whisker plots illustrate the distribution of various Alzheimer's Disease biomarkers (Aβ42, Aβ40, Aβ42/Aβ40 ratio, P-tau181, P-tau217, P-tau231, NTA-tau, NfL, and GFAP) across different stages of chronic kidney disease (CKD). The Y-axis represents the biomarker concentrations, while the X-axis shows CKD stages 1, 2, and 3, with grey indicating CKD stage 1, orange for stage 2, and blue for stage 3. Each box plot displays the median (horizontal line), the interquartile range (IQR) from the 1st quartile (Q1) to the 3rd quartile (Q3), whiskers extending to 1.5 times the IQR, and outliers shown as points beyond the whiskers. Biomarker levels tend to increase with CKD severity, with the highest levels generally observed in CKD stage 3. For a detailed comparison between groups, see Supplementary Table 1

Plasma Aβ concentrations altered across the various CKD stages. Additionally, adjustments for age, sex, and amyloid status were carefully applied to account for potential confounding factors. Aβ40 levels were significantly elevated in CKD stage 3 (median (Q1–Q3): 124.00 (113–143) pg/mL, *p* < 0.001), followed by stage 2 (median (Q1–Q3): 93.80 (84.8–105) pg/mL, *p* = 0.002), and stage 1 (median (Q1–Q3): 89.60 (77.2–100) pg/mL, *p* = 0.001) (Table 2, Supplementary Table 1, Fig. 1). Similarly, Aβ42 concentrations were higher in CKD stage 3 (median (Q1–Q3): 8.50 (6.76–10.3) pg/mL, *p* = 0.004) compared to stage 1 (median (Q1–Q3): 6.17 (4.86–7.15) pg/mL, *p* = 0.021). However, no statistically significant difference was observed between stages 2 and 3 (*p* = 0.076). Furthermore, the Aβ42/Aβ40 ratio did not show any statistically significant differences across CKD stages (*p* > 0.05) (Table 2, Supplementary Table 1, Fig. 1).

All plasma p-tau species displayed elevated concentrations in CKD stage 3 compared to earlier CKD stages, but none of these differences reached statistical significance after adjustment for age, sex, and amyloid status. P-tau181 levels were highest in stage 3 (median (Q1–Q3): 11.60 (10.5–15.5) pg/mL), followed by stage 2 (median (Q1–Q3): 8.46 (6.73–12.5) pg/mL), and stage 1 (median (Q1–Q3): 7.98 (5.62–11.9) pg/mL). However, the differences between stages did not achieve statistical significance. Both p-tau217 and p-tau231 levels followed a similar trend, with higher median values in stage 3 (*p*-tau217: 0.57 pg/mL; p-tau231: 23.80 pg/mL) compared to earlier stages, though these differences also failed to reach statistical significance after adjusting for age, sex, and amyloid status (*p* > 0.05) (Table 2, Supplementary Table 1, Fig. 1). Plasma NTA-tau levels also showed a slight increase in CKD stage 3 (median 0.28 pg/mL) compared to stages 1 and 2. However, these differences were not statistically significant (*p* > 0.05), even after adjusting for age, sex, and amyloid status (Table 2, Supplementary Table 1, Fig. 1).

NfL levels demonstrated a significant increase in CKD stage 3 (median (Q1–Q3): 40.40(31.7–48.7) pg/mL, *p* < 0.001), with progressively lower levels in stage 2 (median (Q1–Q3): 26.80 (18.5–32.7) pg/mL, *p* < 0.001) and stage 1 (median (Q1–Q3): 18.80 (14.4–27.1 pg/mL, *p* = 0.001). In contrast, GFAP levels did not show any statistically significant differences between CKD stages (*p* > 0.05), even after adjusting for age, sex, and amyloid status (Table 2, Supplementary Table 1, Fig. 1).

We also compared plasma biomarkers between individuals with normal renal function and those with mild renal impairment, adjusting for age, sex, and amyloid status. Statistically significant differences were observed for plasma Aβ42, Aβ40, and NfL (*p* ≤ 0.05), while other biomarkers did not show significant differences after adjusting for age, sex, and amyloid status (Supplementary Table 2, Supplementary Fig. 2).

### The relationship between eGFR and plasma AD biomarker concentrations

## Associations between eGFR and plasma biomarkers

**Fig. 2:**
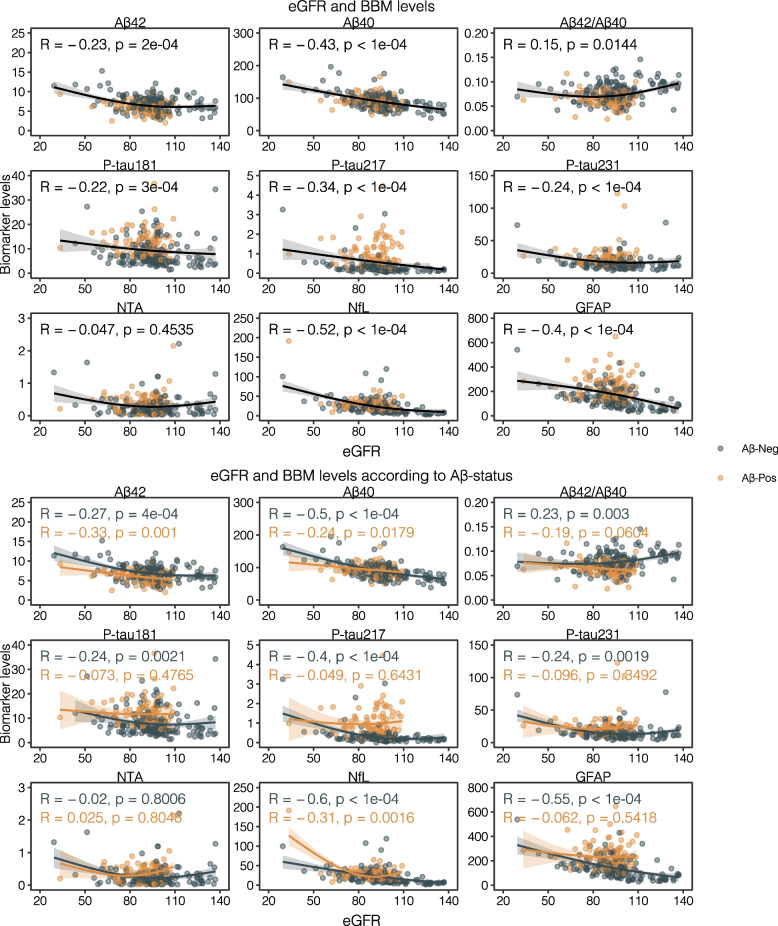
Scatter Plots Depicting the Relationship Between eGFR and Plasma Biomarkers in Aβ-Positive and Aβ-Negative Individuals **a** eGFR and BBM levels. Scatter plots in the upper half of the layout showing the relationship between eGFR and Alzheimer's disease blood-based biomarkers (BBM) across the entire cohort. The plots illustrate an inverse trend between eGFR and several biomarkers, including Aβ42, Aβ40, p-tau181, p-tau217, p-tau231, NfL, and GFAP. A positive trend is observed for the Aβ42/Aβ40 ratio. NTA-tau does not appear to show a significant relationship with eGFR. Grey points represent Aβ-negative individuals, while orange points represent Aβ-positive individuals. Non-linear regression lines, represented by solid curves, are fitted using a generalized additive model with cubic splines (3 knots), and the shaded areas around them indicate 95% confidence intervals. Spearman’s rho is used to numerically represent the associations. **b** – eGFR and BBM levels according to Aβ status. Scatter plots illustrating the relationship between eGFR and Alzheimer's disease biomarkers, stratified by amyloid status (Aβ-negative and Aβ-positive). In the Aβ-negative group (grey points), an inverse relationship is seen between eGFR and several biomarkers, including Aβ42, Aβ40, p-tau181, p-tau217, p-tau231, NfL, and GFAP, with a positive relationship for the Aβ42/Aβ40 ratio. In the Aβ-positive group (orange points), the relationship between eGFR and these biomarkers is generally weaker, with fewer noticeable trends compared to the Aβ-negative group. Non-linear regression lines, represented by solid curves, are fitted using a generalized additive model with cubic splines (3 knots), and the shaded areas around them indicate 95% confidence intervals. Spearman’s rho is used to numerically represent the associations

A significant inverse correlation was observed between eGFR and multiple AD biomarkers, including Aβ42 (rho = -0.23, *p* = 2e-04), Aβ40 (rho = -0.43, *p* < 1e-04), p-tau181 (rho = -0.22, *p* = 3e-04), p-tau217 (rho = -0.34, *p* < 1e-04), p-tau231 (rho = -0.24, *p* < 1e-04), NfL (rho = -0.52, *p* < 1e-04), and GFAP (rho = -0.40, *p* < 1e-04) (Fig. 2a). In contrast, a positive correlation was observed between the Aβ42/Aβ40 ratio and eGFR (rho = 0.15, *p* = 0.0144). However, NTA-tau did not show a significant correlation with eGFR (rho = -0.047, *p* = 0.4535) (Fig. 2a).

When dividing the cohort into two subgroups based on amyloid status (Aβ-negative and Aβ-positive), distinct patterns emerged. In the Aβ-negative group, significant inverse correlations were observed between eGFR and several biomarkers, including Aβ42 (rho = -0.27, *p* = 4e-04), Aβ40 (rho = -0.5, *p* < 1e-04), *p*-tau181 (rho = -0.24, *p* = 0.0021), p-tau217 (rho = -0.4, *p* < 1e-04), p-tau231 (rho = -0.24, *p* = 0.0019), NfL (rho = -0.6, *p* < 1e-04), and GFAP (rho = -0.55, *p* < 1e-04) (Fig. 2). Additionally, the Aβ42/Aβ40 ratio showed a positive correlation with eGFR (rho = 0.23, *p* = 0.003). No significant correlations were found for NTA-tau (rho = -0.02, *p* = 0.8006) (Fig. 2b).

In contrast, in the Aβ-positive group, fewer significant correlations were observed. For Aβ40, a weaker but still significant inverse correlation was seen (rho = -0.24, *p* = 0.0179). While the correlation for Aβ42 (rho = -0.33, *p* = 0.001) was statistically significant, the Aβ42/Aβ40 ratio (rho = -0.19, *p* = 0.0604) did not reach the threshold for significance. Among the other biomarkers, NfL demonstrated a statistically significant inverse correlation with eGFR (rho = -0.31, *p* = 0.0016), whereas no significant correlations were found for p-tau181 (rho = -0.073, *p* = 0.4765), p-tau217 (rho = -0.049, *p* = 0.6431), p-tau231 (rho = -0.096, *p* = 0.3492), and GFAP (rho = -0.062, *p* = 0.5418), indicating a weaker relationship between these biomarkers and eGFR in this group. As previously observed, NTA-tau did not show significant correlations with eGFR (rho = 0.025, *p* = 0.8048) (Fig. 2b).

## Multivariable regression analysis of eGFR and plasma biomarkers

**Fig. 3:**
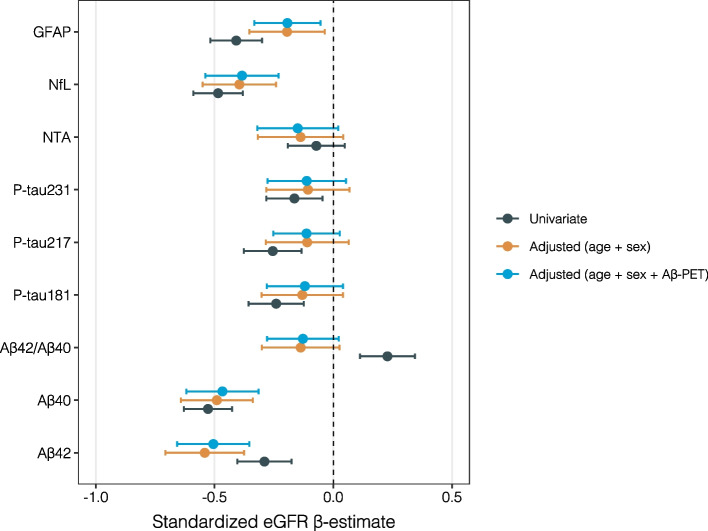
Impact of eGFR on Alzheimer's Disease Biomarkers: Univariate and Multivariable Analyses. Forest plot displaying the standardized β-estimates of eGFR across various Alzheimer's Disease biomarkers, including Aβ42, Aβ40, Aβ42/Aβ40 ratio, P-tau217, P-tau231, NfL, and GFAP. The estimates are shown for three models: univariate (black), adjusted for age and sex (orange), and adjusted for age, sex, and Aβ-PET status (blue). Negative β-estimates indicate an inverse relationship between eGFR and biomarker levels

A significant inverse association was observed between standardized eGFR and multiple plasma biomarkers across different models. In the univariate analysis, lower eGFR was associated with the levels of Aβ42 (β = -0.29, 95% CI: -0.40 to -0.18, *p* < 0.001), Aβ40 (β = -0.53, 95% CI: -0.63 to -0.43, *p* < 0.001), p-tau217 (β = -0.25, 95% CI: -0.38 to -0.13, *p* < 0.001), p-tau231 (β = -0.16, 95% CI: -0.28 to -0.05, *p* = 0.007), *p*-tau181 (β = -0.24, 95% CI: -0.36 to -0.12, *p* < 0.001), NfL (β = -0.49, 95% CI: -0.59 to -0.38, *p* < 0.001), and GFAP (β = -0.41, 95% CI: -0.52 to -0.30, *p* < 0.001). However, the Aβ42/Aβ40 ratio exhibited a positive association with eGFR (β = 0.23, 95% CI: 0.11 to 0.34, *p* < 0.001), while NTA-tau did not show a significant relationship with eGFR (β = -0.07, 95% CI: -0.19 to 0.05, *p* = 0.24) (Table 3, Fig. 3).

**Table 3:** Standardized eGFR β-Estimates for Plasma Alzheimer's Disease Biomarkers: Univariate and Multivariable Models

Biomarker	Model	β Estimate	95% CI (Lower)	95% CI (Upper)	*p*-value
Plasma Aβ42	Univariate	-0.29	-0.40	-0.18	9.92e-7
Plasma Aβ42	Adjusted (age + sex)	-0.54	-0.71	-0.38	5.86e-10
Plasma Aβ42	Adjusted (age + sex + Aβ-PET)	-0.51	-0.66	-0.35	3.11e-10
Plasma Aβ40	Univariate	-0.53	-0.63	-0.43	3.98e-21
Plasma Aβ40	Adjusted (age + sex)	-0.49	-0.64	-0.34	7.93e-10
Plasma Aβ40	Adjusted (age + sex + Aβ-PET)	-0.47	-0.62	-0.32	4.75e-9
Plasma Aβ42/40	Univariate	0.23	0.11	0.34	< 0.001
Plasma Aβ42/40	Adjusted (age + sex)	-0.14	-0.30	0.03	0.100
Plasma Aβ42/40	Adjusted (age + sex + Aβ-PET)	-0.13	-0.28	0.02	0.097
Plasma p-tau181	Univariate	-0.24	-0.36	-0.12	5.91e-5
Plasma p-tau181	Adjusted (age + sex)	-0.13	-0.30	0.04	0.136
Plasma p-tau181	Adjusted (age + sex + Aβ-PET)	-0.12	-0.28	0.04	0.143
Plasma p-tau217	Univariate	-0.25	-0.38	-0.13	4.87e-5
Plasma p-tau217	Adjusted (age + sex)	-0.11	-0.28	0.06	0.216
Plasma p-tau217	Adjusted (age + sex + Aβ-PET)	-0.11	-0.25	0.03	0.113
Plasma p-tau231	Univariate	-0.16	-0.28	-0.05	0.007
Plasma p-tau231	Adjusted (age + sex)	-0.11	-0.28	0.07	0.232
Plasma p-tau231	Adjusted (age + sex + Aβ-PET)	-0.11	-0.28	0.05	0.183
Plasma NTA-tau	Univariate	-0.07	-0.19	0.05	0.242
Plasma NTA-tau	Adjusted (age + sex)	-0.14	-0.32	0.04	0.132
Plasma NTA-tau	Adjusted (age + sex + Aβ-PET)	-0.15	-0.32	0.02	0.085
Plasma NfL	Univariate	-0.49	-0.59	-0.38	1.21e-17
Plasma NfL	Adjusted (age + sex)	-0.40	-0.55	-0.24	8.64e-7
Plasma NfL	Adjusted (age + sex + Aβ-PET)	-0.38	-0.54	-0.23	1.55e-6
Plasma GfAP	Univariate	-0.41	-0.52	-0.30	1.66e-12
Plasma GfAP	Adjusted (age + sex)	-0.19	-0.35	-0.04	0.016
Plasma GfAP	Adjusted (age + sex + Aβ-PET)	-0.19	-0.33	-0.05	0.007

This table presents the β-estimates, 95% confidence intervals (CI), and p-values for the association between standardized eGFR and various plasma AD biomarkers, including Aβ42, Aβ40, p-tau217, p-tau231, NfL, GFAP, and the Aβ42/Aβ40 ratio. The results are shown for three different models: univariate (no adjustments), adjusted for age and sex, and adjusted for age, sex, and Aβ-PET status. The p-values indicate the statistical significance of these associations, with values below 0.05 considered significant

Abbreviations: *eGFR* estimated glomerular filtration rate, *Aβ42* amyloid-beta 42, *Aβ40* amyloid-beta 40, *p-tau181* phosphorylated tau at threonine 181, *p-tau217* phosphorylated tau at threonine 217, *p-tau231* phosphorylated tau at threonine 231, *NTA-tau* N-terminal containing tau fragments, *NFL* Neurofilament light chain, *GFAP* Glial fibrillary acidic protein, *Aβ-PET* Amyloid-beta positron emission tomography

After adjusting for age and sex, the associations remained significant for Aβ42 (β = -0.54, 95% CI: -0.71 to -0.38, *p* < 0.001), Aβ40 (β = -0.49, 95% CI: -0.64 to -0.34, *p* < 0.001). GFAP (β = -0.19, 95% CI: -0.35 to -0.04, *p* = 0.016) remained significant after adjustment. The associations with p-tau217 (β = -0.11, 95% CI: -0.28 to 0.06, *p* = 0.216), p-tau231 (β = -0.11, 95% CI: -0.28 to 0.07, *p* = 0.23), and p-tau181 (β = -0.13, 95% CI: -0.30 to 0.04, *p* = 0.14) weakened and became non-significant. NfL showed a weaker but still significant association with eGFR (β = -0.40, 95% CI: -0.55 to -0.24, *p* < 0.001), while the Aβ42/Aβ40 ratio also became non-significant (β = -0.14, 95% CI: -0.30 to 0.02, *p* = 0.10). NTA-tau remained non-significant (β = -0.14, 95% CI: -0.32 to 0.04, *p* = 0.13) (Table 3, Fig. 3).

After further adjustment for Aβ-PET status, the associations for Aβ42 (β = -0.51, 95% CI: -0.66 to -0.35, *p* < 0.001) and Aβ40 (β = -0.47, 95% CI: -0.62 to -0.32, *p* < 0.001) remained robust, while GFAP continued to show a significant association (β = -0.19, 95% CI: -0.33 to -0.05, *p* = 0.007). The association between eGFR and NfL remained significant (β = -0.38, 95% CI: -0.54 to -0.23, *p* < 0.001), while *p*-tau217 (β = -0.11, 95% CI: -0.25 to 0.03, *p* = 0.11), p-tau231 (β = -0.11, 95% CI: -0.28 to 0.05, *p* = 0.18), and p-tau181 (β = -0.12, 95% CI: -0.28 to 0.04, *p* = 0.14) were not significantly associated. The Aβ42/Aβ40 ratio remained non-significant (β = -0.13, 95% CI: -0.28 to 0.02, *p* = 0.10). NTA-tau remained non-significant across all models (β = -0.15, 95% CI: -0.32 to 0.02, *p* = 0.09) (Table 3, Fig. 3).

To adjust for any skewness in the data, sensitivity analyses were made log-transforming eGFR and biomarker levels, with similar results throughout for all biomarkers (Supplementary Fig. 4, 5).

## Assessing the incremental value of eGFR in predicting Aβ-PET positivity using plasma biomarkers

To further assess the predictive value of plasma biomarkers for Aβ-PET positivity, logistic regression analyses were conducted to evaluate the AUC and AIC across different models. The AUC for each biomarker was calculated for univariate models, models adjusted for age and sex, and fully adjusted models that included eGFR.

**Fig. 4:**
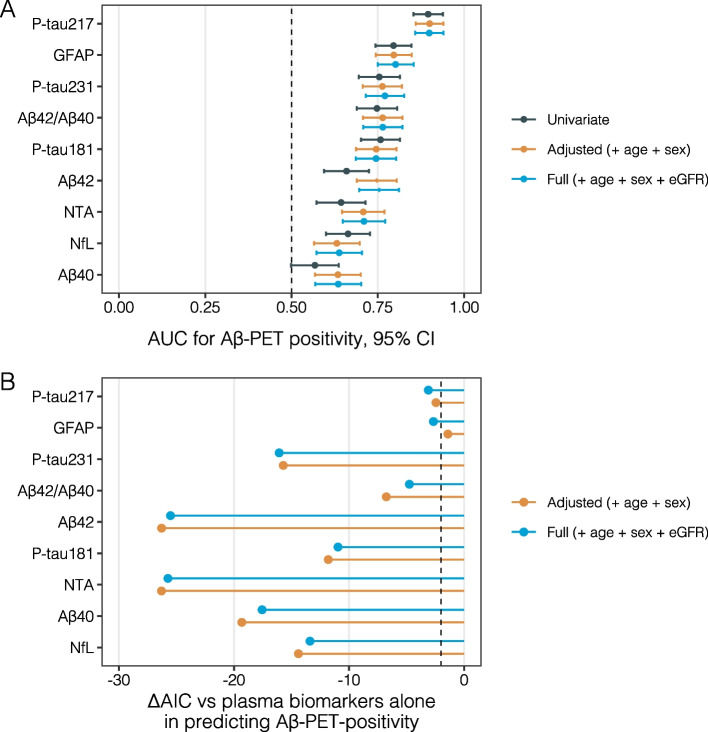
Evaluation of Model Performance for Aβ-PET Positivity: Influence of eGFR on AUC and AIC. **A** Area Under the Curve (AUC) values for predicting Aβ-PET positivity using various AD biomarkers across three models: univariate, adjusted for age and sex, and fully adjusted (age + sex + eGFR). The dashed vertical line at 0.50 represents the threshold where the model has no discriminatory ability (essentially, no better than random chance). A higher AUC value indicates better predictive accuracy for Aβ-PET positivity. **B** ΔAIC values comparing the improvement in model fit by adding eGFR to the age + sex model. The analysis shows that while eGFR adds some predictive value, the improvement in model performance is limited compared to the contribution of age and sex. The dashed vertical line corresponds to the ΔAIC of -2, commonly used in the literature as the minimal difference in AIC to denote statistical significance

The ΔAIC values revealed that while the addition of age and sex to plasma biomarkers significantly improved model fit, the inclusion of eGFR had minimal impact. For instance, in predicting Aβ-PET positivity, the AIC for the Aβ42 improved by 26.3 points when age and sex were added, but further inclusion of eGFR led to only a slight change (ΔAIC = 0.78). Similarly, for Aβ40, the AIC improved by 19.3 points with age and sex, while adding eGFR resulted in a negligible difference (ΔAIC = 1.76). For p-tau217, the improvement in AIC was 2.44 points after adjusting for age and sex, with eGFR contributing minimally to model improvement (ΔAIC = -0.66). In the case of NfL, the AIC improved by 14.4 points with age and sex, while the inclusion of eGFR made no notable impact (ΔAIC = 1.0). Similarly, NTA-tau showed an improvement in AIC by 26.3 points with age and sex, with a negligible change when eGFR was added (ΔAIC = 0.56) (Table 4, Fig. 4).

**Table 4:** Summary of AUC and AIC metrics for plasma biomarkers in predictive models of Aβ-PET positivity

			**Confidence Interval 95%**						
**Biomarker**	**Model**	**AUC**	**Lower Limit**	**Upper Limit**	**AIC**	**AICc**	***p* value (de long)**	**ΔAICc**	**ΔAICc with eGFR**
Plasma Aβ42	Univariate	0.659	0.594	0.724	336.07	336.16		0	
Plasma Aβ42	Adjusted (+ age + sex)	0.747	0.689	0.805	309.63	309.86	0.001	-26.30	
Plasma Aβ42	Full (+ age + sex + eGFR)	0.754	0.696	0.811	310.32	310.64	0.000	-25.52	0.78
Plasma Aβ40	Univariate	0.568	0.498	0.637	356.74	356.83		0	
Plasma Aβ40	Adjusted (+ age + sex)	0.634	0.568	0.701	337.28	337.51	0.137	-19.32	
Plasma Aβ40	Full (+ age + sex + eGFR)	0.635	0.569	0.702	338.95	339.27	0.126	-17.56	1.76
Plasma Aβ42/40	Univariate	0.747	0.689	0.806	311.21	311.30		0	
Plasma Aβ42/40	Adjusted (+ age + sex)	0.764	0.707	0.821	304.31	304.54	0.235	-6.77	
Plasma Aβ42/40	Full (+ age + sex + eGFR)	0.764	0.707	0.821	306.22	306.54	0.225	-4.76	2.01
Plasma p-tau181	Univariate	0.758	0.701	0.814	329.48	329.57		0	
Plasma p-tau181	Adjusted (+ age + sex)	0.745	0.687	0.804	317.53	317.77	0.513	-11.81	
Plasma p-tau181	Full (+ age + sex + eGFR)	0.745	0.686	0.803	318.29	318.61	0.526	-10.96	0.85
Plasma p-tau217	Univariate	0.896	0.853	0.939	236.73	236.83		0	
Plasma p-tau217	Adjusted (+ age + sex)	0.900	0.860	0.940	234.13	234.38	0.613	-2.44	
Plasma p-tau217	Full (+ age + sex + eGFR)	0.899	0.858	0.940	233.36	233.72	0.781	-3.11	-0.66
Plasma p-tau231	Univariate	0.754	0.694	0.814	323.00	323.10		0	
Plasma p-tau231	Adjusted (+ age + sex)	0.763	0.707	0.820	307.14	307.38	0.673	-15.72	
Plasma p-tau231	Full (+ age + sex + eGFR)	0.771	0.715	0.826	306.70	307.02	0.482	-16.07	-0.35
Plasma NTA-tau	Univariate	0.644	0.573	0.714	342.93	343.02		0	
Plasma NTA-tau	Adjusted (+ age + sex)	0.708	0.646	0.770	316.48	316.71	0.084	-26.30	
Plasma NTA-tau	Full (+ age + sex + eGFR)	0.710	0.648	0.772	316.95	317.28	0.083	-25.74	0.56
Plasma NfL	Univariate	0.664	0.600	0.727	351.77	351.86		0	
Plasma NfL	Adjusted (+ age + sex)	0.631	0.565	0.697	337.23	337.46	0.290	-14.40	
Plasma NfL	Full (+ age + sex + eGFR)	0.638	0.572	0.704	338.14	338.46	0.421	-13.39	1.00
Plasma GfAP	Univariate	0.795	0.743	0.847	294.03	294.12		0	
Plasma GfAP	Adjusted (+ age + sex)	0.796	0.744	0.848	292.48	292.71	0.938	-1.41	
Plasma GfAP	Full (+ age + sex + eGFR)	0.801	0.749	0.854	291.14	291.46	0.623	-2.67	-1.26

*eGFR* estimated glomerular filtration rate, *Aβ42* Amyloid-beta 42, *Aβ40* Amyloid-beta 40; *p-tau181* phosphorylated tau at threonine 181, *p-tau217* phosphorylated tau at threonine 217, *p-tau231* phosphorylated tau at threonine 231, *NTA-tau* N-terminal containing tau fragments, *NFL* Neurofilament light chain, *GFAP* Glial fibrillary acidic protein, *Aβ-PET* Amyloid-beta positron emission tomography

This table presents a detailed comparison of area under the curve (AUC), Akaike Information Criterion (AIC), and ΔAIC values across different predictive models for Aβ-PET positivity, using various plasma biomarkers including Aβ42, Aβ40, the Aβ42/40 ratio, P-tau181, P-tau217, P-tau231, and NfL. The models considered include univariate models (plasma biomarkers alone), models adjusted for age and sex, and fully adjusted models that also include eGFR

For each biomarker, the AUC values are provided along with their confidence intervals (95% CI) to indicate the model's discriminative ability, AIC and AICc values are presented to assess model fit, with ΔAIC showing the improvement gained by adding age, sex, and eGFR to the models. The *p*-values from DeLong's test for comparing AUCs are also included to assess the statistical significance of the differences between models

In terms of AUC, the addition of eGFR resulted in minimal improvements in clinical discrimination. For instance, the AUC for the Aβ42/40 ratio increased from 0.75 in the univariate model to 0.76 after adjusting for age and sex, with no further improvement when eGFR was added (AUC = 0.76). Similarly, for Aβ40, the AUC increased from 0.57 to 0.63 with age and sex and marginally improved to 0.64 with eGFR. Plasma p-tau217, the AUC increased slightly from 0.896 in the univariate model to 0.900 after adjusting for age and sex, with a negligible change to 0.899 when eGFR was added. For plasma NfL, the AUC decreased from 0.664 in the univariate model to 0.631 after adjusting for age and sex, with a slight increase to 0.638 when eGFR was added. Finally, NTA-tau's AUC improved from 0.64 to 0.71 after adjusting for age and sex, with no further improvement when eGFR was added (AUC = 0.71) (Table 4, Fig. 4).

These findings suggest that while age and sex substantially improve the predictive power of plasma biomarkers for Aβ-PET positivity, the inclusion of eGFR adds little to no further benefit in terms of model fit or discrimination ability across all evaluated biomarkers, including p-tau181 and NTA-tau. Examining the functional relationship between biomarkers, eGFR, and the risk of Aβ-PET positivity using model-derived probabilities from each full model revealed that biomarkers with strong discriminative power for Aβ-status (e.g., p-tau217) exhibited sigmoidal patterns approaching probabilities near 0 and 1 (Supplementary Fig. 3). In contrast, eGFR consistently displayed an approximately horizontal functional form across all models, underscoring its low impact on the biomarkers' predictive ability for Aβ-status in this sample.

## Discussion

This study examined the relationship between kidney function, as measured by eGFR, and the concentrations of peripheral AD biomarkers, as well as the role of kidney impairment in predicting amyloid positivity. The levels of all AD biomarkers were increased the most in CKD stage 3, followed by CKD stage 2 and CKD stage 1. Furthermore, the AD biomarkers investigated were initially found to be significantly associated with eGFR. However, after adjusting for age, sex, and Aβ positivity, the associations for the Aβ42/40 ratio, p-tau181, p-tau217, and p-tau231 were no longer significant. Plasma NTA-tau remained non-significantly associated with eGFR across all models. Despite these adjustments, significant associations remained for other biomarkers, including NfL, Aβ42, Aβ40 and GFAP. We also observed that adding eGFR to the prediction models for Aβ positivity did not enhance their performance, whereas the inclusion of age and sex significantly improved model fit when combined with plasma biomarkers. Taken together, our study provides evidence that blood AD biomarkers increase as kidney function declines and are associated with eGFR. However, when incorporated into amyloid positivity prediction models, these biomarkers did not improve the models' predictive performance.

Blood-based biomarkers hold significant potential for diagnosing and predicting the progression of AD [12]. However, before they can be widely adopted, it is crucial to thoroughly understand the biological and technical factors that could compromise their diagnostic accuracy. Moreover, for inclusion in clinical trials for AD, biomarkers should accurately identify participants who are in the preclinical phase of the disease [7, 37, 38] and track treatment efficacy [19, 20]. Additionally, any drifts in biomarkers that are independent of amyloid pathology could lead to misclassification of the current pathology [8, 9, 39], inappropriate selection of participants for clinical trials, and misinterpretation of treatment responses [7]. Recently, the Global CEO Initiative on Alzheimer’s Disease convened a blood biomarker (BBM) workgroup and made recommendations on the acceptable performance of BBM tests for amyloid pathology [40]. The importance of using a two cut-off approach was also emphasized, along with the observation that BBMs are associated with kidney dysfunction[40, 41]. Notably, BBM ratios, such as Aβ42/40 and p-tau217 to non-phosphorylated tau, were highlighted for their consistent performance and reduced susceptibility to kidney dysfunction [40], as demonstrated in several previous publications [25, 26, 42]. In the recently revised criteria for the biological diagnosis and staging of AD, NfL and GFAP are not included in the core biomarker category to aid in diagnosis [43]. However, they are mentioned as useful for staging, prognosis, and as indicators of biological treatment effects, serving as markers of neurodegeneration and inflammation, respectively [43]. Therefore, it is important to determine the effect of kidney dysfunction on these biomarkers, along with the core biomarkers.

Given this context, it is important to note that CKD represents a global public health burden, with early stages often being asymptomatic. As a result, CKD is commonly diagnosed at later stages. The global estimated prevalence of CKD is 13.4% [44]. Studies have reported that in the general population, particularly among older adults, the rate of decline in eGFR can range from about 0.75 to 1.2 mL/min/1.73 m^2^ per year [45–47]. Given that the most common form of sporadic AD typically occurs around the age of 60 [48], the influence of CKD should be considered. Therefore, previous studies have investigated the effect of kidney impairment on AD biomarkers [25, 26, 49]. Our study adds to this growing body of evidence and is in line with previous studies that investigated the effect of kidney dysfunction on plasma biomarkers via eGFR or creatinine [22, 25, 26, 31, 42, 49, 50]. For example, among NfL, GFAP, and p-tau217, NfL showed the strongest association with eGFR, followed by GFAP and p-tau217. Even after adjusting for age, sex, and amyloid positivity, plasma NfL and GFAP remained significantly associated with kidney impairment, suggesting that the influence of kidney dysfunction should be considered when assessing neuronal injury and astrocyte activation in patients with AD. These findings are consistent with previous studies [25–27], underscoring the importance of considering kidney function as a factor that can affect biomarker interpretation [26]. In our study, we used eGFR instead of creatinine, and this methodological difference, along with our relatively smaller sample size, may explain any discrepancies in our findings. Janalidze et al. have shown that using ratios for p-tau species can mitigate the influence of kidney impairment on blood p-tau levels [42]. Although plasma NTA-tau did not show a significant association with eGFR, we observed similar findings for the p-tau species in our study. Before adjusting for covariates, p-tau181, p-tau217, and p-tau231 were significantly associated with eGFR. However, after adjusting for covariates, these associations were no longer significant. Building on these findings, incorporating covariates, such as eGFR, in a two-step model of p-tau217 could reduce the misclassification rate, particularly in intermediate categories; however, further research is needed in this area.

Building on these observations, when the biomarkers were analyzed separately by amyloid status and correlation analysis was performed between eGFR and plasma biomarkers, the significance disappeared for the Aβ42/40 ratio, p-tau species, and GFAP in Aβ-positive cases. However, significant correlations persisted for Aβ42, Aβ40, and NfL within this group. Notably, no significant correlations were observed for NTA-tau in either Aβ-positive or Aβ-negative individuals.It is known that in individuals without pathological brain amyloid accumulation, plasma Aβ levels are predominantly derived from peripheral sources, such as platelet production, and are influenced by clearance mechanisms involving the liver, spleen, and kidneys [51, 52]. This peripheral origin likely explains the stronger correlation observed between eGFR and plasma Aβ levels, as renal function plays a key role in the clearance of these peptides. However, when examining the Aβ42/Aβ40 ratio, the significance of correlations with eGFR diminishes, suggesting that renal function has a lesser impact on this biomarker compared to individual Aβ species. This observation aligns with findings that the Aβ42/Aβ40 ratio is less influenced by peripheral factors and more indicative of CNS pathology [53]. For the three p-tau species, the disappearance of significant correlations in Aβ-positive cases may be attributed to an active tubular secretion or renal clearance mechanism that becomes impaired in the presence of amyloid pathology. Further studies are necessary to investigate and confirm this hypothesis. Additionally, for NfL (where a minor peripheral contribution cannot be excluded [54]) and GFAP, their plasma levels are primarily driven by CNS pathology rather than peripheral clearance mechanisms. This may also apply to other biomarkers, where overproduction or excessive release from the CNS could exceed the renal clearance capacity. Such dynamics likely explain the absence of significant correlations with eGFR, as renal function appears to exert minimal influence on their plasma concentrations in the Aβ-positive group.

Additionally, we compared different predictive models, including a univariate model, an adjusted model (age + sex), and a full model (age + sex + eGFR), to assess whether the inclusion of eGFR alongside other covariates could enhance the prediction of amyloid positivity. We then evaluated model fit by examining the impact of adding eGFR to the age + sex model using AIC. The findings revealed that eGFR does not contribute to the prediction of Aβ-PET positivity, its effect is relatively modest compared to that of age and sex. Similar to the existing literature [55], where most studies did not include eGFR in their prediction models [8, 56], our findings also underscore a more dominant role of AD pathology and demographic factors over biomarker levels, given no improvement in model fit nor in discrimination were observed by adding eGFR. These findings indicate that this outcome is affected by the relationship between age, sex and AD pathology status, reinforcing the limited added value of eGFR in this context. Moreover, in the future, as AD biomarkers are implemented in routine practice—whether as stand-alone tests (e.g., p-tau217) or as part of multi-test panels (e.g., NfL, GFAP)—accounting for demographic factors will likely require the development of cutoff matrices based on sex and age. Alternatively, the use of well-calibrated risk prediction models may also solve such issue, since they provide a covariate-adjusted probability value to which a more intuitive cut-off can be applied without needing sub-stratifying patient populations. Overall, these results indicate that while age and sex significantly add predictive value for Aβ-PET positivity when combined with plasma biomarkers, eGFR may be less relevant.

This study has several strengths and limitations. First, we investigated a broad range of blood biomarkers and their associations with kidney function, which had not yet been done in such extent. Moreover, we utilized eGFR to objectively assess renal function and its relationship with plasma biomarkers, rather than relying on self-reported kidney dysfunction or a medical history of CKD. Furthermore, our study included prediction models where eGFR, as a proxy for kidney impairment, was incorporated into the analyses. However, due to the cross-sectional design of the study, we cannot infer a causal relationship between decreased renal function and elevated plasma biomarker levels. Previous studies [57, 58] have suggested that a possible kidney-brain axis may contribute to the development of dementia. Nonetheless, as mentioned earlier, we are unable to draw such causal conclusions from our findings. On top of that, incorporating cystatin C into the eGFR calculation could provide a more accurate estimation, as demonstrated in a large Swedish study [59], particularly in older populations. However, since cystatin C data was not available in this study, we were unable to assess the potential influence of cystatin C on eGFR calculation and its impact on the prediction models. Additionally, the small number of participants in CKD stage 3 could have led to an underestimation of the observed effects. An additional limitation relates to the lack of racial and ethnic diversity in our study population, with most participants being White. This restricts the extrapolation of our findings to more diverse populations. Future studies should aim to include participants from diverse racial and ethnic backgrounds to enhance the generalizability of the findings. Moreover, the limited number of participants in CKD stage 3 or more advanced stages, with most participants in the current study categorized as having mild CKD, restricts generalizability of the results. Therefore, the findings on lack of a clinically relevant impact of renal function over AD blood biomarkers should be considered within the spectrum of mild-to-moderate renal function impairment. Future studies should include more individuals with severe CKD (stage 3 and beyond) and focus on pre-symptomatic AD to determine whether severely impaired kidney function impacts p-tau217-supported clinical diagnosis. Finally, while our findings were based on a well-defined cohort, more real-world effects might have been observed if these variables were investigated in a community-based cohort.

In summary, this study offers a detailed examination of how kidney function affects blood biomarker levels, utilizing a wide array of plasma biomarkers in a well-defined cohort, with most participants falling within the normal renal function to mild renal impairment range. Although all the biomarkers studied were initially associated with eGFR, further adjustments for additional covariates revealed the need for more precise evaluations. Future studies with population-based cohorts are necessary to explore the relationship between renal function and other AD plasma biomarkers, considering a broader range of potential confounders. These studies should aim to validate the findings across diverse populations and incorporate covariates, such as eGFR, into two-step decision models. Ultimately, diagnostic and prognostic algorithms for AD based on plasma biomarkers may not be improved by adding eGFR into the models despite the apparent increase in biomarker levels as kidney function declines.

## Supplementary Information

Supplementary Material 1.

## Data Availability

Data from the TRIAD cohort supporting this study’s findings are available upon reasonable request to the corresponding author and subject to review by McGill University for intellectual property or confidentiality considerations. Anonymized data may be shared with qualified academic investigators for result replication under a material transfer agreement. Due to privacy concerns and the risk of compromising research participants' confidentiality, data are not publicly available.

## Abbreviations

AD: Alzheimer’s disease
PET: Positron emission tomography
CSF: Cerebrospinal fluid
Aβ: Amyloid-beta
p-tau: Phosphorylated tau
NTA-tau: N-terminal containing tau fragments
NfL: Neurofilament light
GFAP: Glial fibrillary acidic protein
DMT: Disease-modifying therapies
eGFR: Estimated glomerular filtration rate
B2M: β2-Microglobulin
CKD: Chronic kidney disease
TRIAD: Translational Biomarkers in Aging and Dementia
CDR: Clinical Dementia Rating
MMSE: Mini-Mental State Examination
MRI: Magnetic resonance imaging
KDIGO-CKD: Kidney Disease: Improving Global Outcomes
AUC: Area under the curve
AIC: Akaike Information Criterion
BBM: Blood biomarker
