# Supplementary material for: The impact of kidney function on Alzheimer’s disease blood biomarkers: implications for predicting amyloid-β positivity
Source: Alzheimers Res Ther. 2025 Feb 19;17:48. doi: 10.1186/s13195-025-01692-z (PMC11837363; doi:10.1186/s13195-025-01692-z)
Supplement: Supplementary file 1 — Supplementary Material 1. [file 13195_2025_1692_MOESM1_ESM.docx]

**Supplementary Materials**

**The Impact of Kidney Function on Alzheimer’s Disease Blood Biomarkers: Implications for Predicting Amyloid-β Positivity**

Burak Arslan^1,14*^, Wagner Brum^1,2*^, Ilaria Pola^1^, Joseph Therriault^3^, Nesrine Rahmouni^3^, Jenna Stevenson^3^, Stijn Servaes^3^, Kübra Tan^1^, Paolo Vitali^3^, Maxime Montembeault^3^, Jesse Klostranec^3^, Arthur C. Macedo^3^, Cecile Tissot^3,4^, Serge Gauthier^3^, Juan Lantero-Rodriguez^1^, Eduardo R. Zimmer^2,5,6,7^, Kaj Blennow^1, 8, 9, 14^, Henrik Zetterberg^1,13,14,15,16,17^, Pedro Rosa-Neto^3^, Andrea L. Benedet^1#^, Nicholas J. Ashton^1,10,11,12^ ^#^

**Affiliations**

^1^Department of Psychiatry and Neurochemistry, Institute of Neuroscience & Physiology, the Sahlgrenska Academy at the University of Gothenburg, Mölndal, Sweden

^2^Graduate Program in Biological Sciences: Biochemistry, Universidade Federal do Rio Grande do Sul (UFRGS), Porto Alegre, Brazil

^3^Translational Neuroimaging Laboratory, McGill University Research Centre for Studies in Aging, Montreal Neurological Institute-Hospital, Douglas Research Institute, Department of Neurology and Neurosurgery, Psychiatry and Pharmacology and Therapeutics, McGill University, Montreal, Canada

^4^ Lawrence Berkeley National Laboratory, Berkeley, CA, USA

^5^Graduate Program in Biological Sciences: Pharmacology and Therapeutics, UFRGS, Porto Alegre, Brazil

^6^ Department of Pharmacology, UFRGS, Porto Alegre, Brazil

^7^McGill Centre for Studies in Aging, McGill University, Montreal, Quebec, Canada

^8^Paris Brain Institute, ICM, Pitié-Salpêtrière Hospital, Sorbonne University, Paris, France

^9^Neurodegenerative Disorder Research Center, Division of Life Sciences and Medicine, and Department of Neurology, Institute on Aging and Brain Disorders, University of Science and Technology of China and First Affiliated Hospital of USTC, Hefei, P.R. China

^10^King's College London, Institute of Psychiatry, Psychology and Neuroscience Maurice Wohl Institute Clinical Neuroscience Institute London UK

^11^Banner Alzheimer's Institute and University of Arizona, Phoenix, AZ, USA

^12^Banner Sun Health Research Institute, Sun City, AZ 85351, USA

^13^Wisconsin Alzheimer’s Institute, School of Medicine and Public Health, University of Wisconsin, Madison, WI, USA

^14^ Clinical Neurochemistry Laboratory, Sahlgrenska University Hospital, Gothenburg, Sweden; ^15^Department of Neurodegenerative Disease, Institute of Neurology, University College London, London, UK

^16^UK Dementia Research Institute, University College London, London, UK

^17^Hong Kong Center for Neurodegenerative Diseases, Hong Kong, China

#Correspondence to:

Dr. Nicholas J. Ashton, Ph.D.

Institute of neuroscience and physiology

Dept. Psychiatry and Neurochemistry

Sahlgrenska Academy at Gothenburg University

Mölndal Hospital, Hus V3, 43180 Mölndal, Sweden

E-mail: nicholas.ashton@gu.se

Tel: +46 73-801-1987

Burak Arslan, MD

Institute of neuroscience and physiology

Dept. Psychiatry and Neurochemistry

Sahlgrenska Academy at Gothenburg University

Mölndal Hospital, Hus V3, 43180 Mölndal, Sweden

E-mail: burak.arslan@gu.se

Tel: +46 723-661433

* Burak Arslan and Wagner S. Brum contributed equally as first authors.

# Andrea L. Benedet and Nicholas J. Ashton contributed equally as senior authors.

**Table of Contents**

Supplementary methods

Supplementary tables

**Supplementary Table 1 . Comparison of Plasma Biomarkers Across CKD Stages with Adjustments for Age, Sex, and Amyloid Status**

**Supplementary Table 2. Comparison of Plasma Biomarkers Between Normal Renal Function and Mild Renal Impairment groups with Adjustments for Age, Sex, and Amyloid Status**

**Supplementary table 3. Participant Characteristics and Plasma Biomarker Levels in Groups with Normal Renal Function and Mild Renal Impairment**

Supplementary figures

**Supplementary Figure 1. Associations between eGFR, age, and sex.**

**Supplementary Figure 2.** **Distribution of Alzheimer’s disease biomarkers across normal renal function and mild renal impairment categories**

**Supplementary Figure 3. Biomarker and eGFR levels in relation to risk of Aβ-positivity based on logistic regression models**

**Supplementary Figure 4. Scatter Plots Depicting the Relationship Between eGFR and Plasma Biomarkers in Aβ-Positive and Aβ-Negative Individuals Using Log-Transformed Data**

**Supplementary Figure 5. Impact of eGFR on Alzheimer's Disease Biomarkers: Univariate and Multivariable Analyses based on Log-Transformed Data**

**Supplementary methods**

**The Translational Biomarkers in Aging and Dementia (TRIAD)**

The Translational Biomarkers in Aging and Dementia (TRIAD) is a longitudinal, observational study focused on biomarkers. It has been approved by both the PET working committee at the Montreal Neurological Institute and the Research Ethics Board at the Douglas Mental Health University Institute. All participants gave written informed consent before enrolling in the study. As part of TRIAD, participants undergo yearly assessments, which include thorough clinical evaluations, neuropsychological testing, and the collection of various biofluids (such as blood, urine, saliva, and cerebrospinal fluid), alongside multiple imaging biomarkers.

This study utilizes cross-sectional data from 242 participants in the TRIAD cohort, all of whom underwent multimodal imaging, including [18F]-AZD4694 Aβ PET scans and magnetic resonance imaging (MRI). A subset of these participants also had cerebrospinal fluid (CSF) biomarkers analyzed, including Aβ42/40, p-tau181, p-tau205, p-tau217, and p-tau231.

Cognitively unimpaired (CU) individuals showed no signs of objective cognitive impairment and were assigned a Clinical Dementia Rating (CDR) score of 0. In contrast, individuals with mild cognitive impairment (MCI) displayed either subjective or objective cognitive impairments and had a CDR score of 0.5. Those diagnosed with dementia had CDR scores of 1 or 2.

Structural MRI data were acquired using a 3T Siemens Magnetom scanner to capture high-resolution T1-weighted images of the entire brain. The images were then processed using the SPM12 segmentation tool and non-linearly registered to the ADNI template through DARTEL, as described in previous studies (1). Hippocampal volume was utilized as a marker to estimate brain atrophy, with measurements adjusted for total intracranial volume (ICV), following the methods outlined in previous research (2). The ICV adjustments were based on baseline data from cognitively unimpaired (CU) participants. Additionally, T1-weighted anatomical images were used for coregistration with PET images.

PET imaging was performed using a Siemens High-Resolution Research Tomograph (HRRT), with imaging conducted approximately 80 days after cerebrospinal fluid sample collection (median = 53 days). For Aβ PET, images were acquired 40–70 minutes after the injection of [18F]-AZD4694. The scans were reconstructed using the ordered subset expectation maximization (OSEM) algorithm into a 4-dimensional volume consisting of 3 frames (3x600s) (2). PET images underwent preprocessing steps, including meninges and skull stripping, followed by both linear and non-linear registration to the ADNI template space. Spatial smoothing was applied to achieve a final resolution of 8 mm full width at half maximum (FWHM). The whole cerebellum gray matter was used as the reference region for [18F]-AZD4694. Global Aβ PET was assessed using the averaged standardized uptake value ratio (SUVR) from regions such as the precuneus, cingulate, inferior parietal, medial prefrontal, lateral temporal, and orbitofrontal cortices. A positivity threshold was set at 1.55 SUVR, corresponding to 24 Centiloids. Aβ positivity was also visually confirmed by two neurologists who were blinded to the participants' clinical diagnoses.

Cerebrospinal fluid samples were obtained using a syringe and transferred into polypropylene tubes for centrifugation at 20°C at 2200g for 10 minutes. After centrifugation, the samples were divided into 1-milliliter aliquots and stored at -80°C until they were analyzed at the Department of Neurochemistry, University of Gothenburg. The levels of CSF p-tau181 and Aβ42/40 were measured using the LUMIPULSE G1200, as outlined in previous studies, while p-tau217 and pTau205 were quantified using an in-house Simoa assay developed at the University of Gothenburg. Plasma biomarkers analysis of the TRIAD study was performed at the Department of Psychiatry and Neurochemistry, University of Gothenburg.

Plasma levels of Aβ42/40, GFAP, and NfL were measured using the commercial Neurology 4-plex E kit (#103670, Quanterix). In-house Simoa assays developed at the University of Gothenburg were used to analyze p-tau181(3), p-tau231 (4) , and NTA-tau (5). Plasma P-tau217 was measured using the ALZpath Simoa assay (6).

**Measurement of Plasma Creatinine**

Plasma creatinine levels were assessed utilizing the Roche Cobas chemistry analyzer by Roche Diagnostics (Indianapolis. IN). The analysis was executed through the enzymatic assay known as creatinine PlusVer.2 (REF 03263991). a method developed by Roche and conducted on the c501 platform. In this enzymatic process, creatininase, creatinase, and sarcosine oxidase collaborate to transform creatinine into glycine, formaldehyde, and hydrogen peroxide. The liberated hydrogen peroxide. catalyzed by peroxidase. reacts with 4-aminophenazone and HTIB to create a quinone imine chromogen. The intensity of color exhibited by this quinone imine chromogen directly corresponds to the concentration of creatinine in the reaction mixture.

The assay features a lower limit of detection (LLoD) of 0.057 mg/dL and a measuring range of 0.057-30.5 mg/dL. All samples were analyzed within a day after the validation of the assay using two levels of quality control (QC) samples: Precinorm PUC – LOT 722562 (value range: 104 mg/dL. with a range of 86-122 mg/dL) and PreciControl ClinChem Multi 2 – LOT 535719 (value range: 3.72 mg/dL. with a range of 3.06-4.38 mg/dL).

**Calculation of Estimated GFR**

The provided coefficients are employed in the following formula for estimating the estimated glomerular filtration rate (eGFR) (7):

**eGFR = μ × min (Scr/κ. 1)^a^_1_ × max (Scr/κ. 1)^a^_2_ × c^Age^ × d [if female]**

Here. κ takes the value of 0.7 for female participants and 0.9 for male participants. Min indicates the minimum of Scr/κ and 1. and max indicates the maximum of Scr/κ and 1. It's important to note that the 2009 and 2012 models were developed in previous studies. Sex differences in the eGFRcr are modeled as sex-specific creatinine coefficients, along with coefficients for female sex. The CKD-EPI stands for Chronic Kidney Disease Epidemiology Collaboration, where Scr represents serum creatinine.

The value of μ is 142 for the 2021 CKD-EPI creatinine (2009 CKD-EPI creatinine fit without race)

, specifically for eGFRcr(AS). The coefficient "a_1_" is applied when creatinine levels are less than or equal to 0.9 mg per deciliter for male participants and 0.7 mg per deciliter for female participants. Conversely, the coefficient "a_2_" is used when creatinine levels exceed 0.9 mg per deciliter for male participants and 0.7 mg per deciliter for female participants. More details regarding these coefficients can be found in the relevant paper (7).

While acknowledging the limitations associated with plasma creatinine and the 24-hour creatinine clearance method, the National Kidney Foundation Disease Outcomes Quality Initiative (K-DOQI) has endorsed the utilization of estimated glomerular filtration rate (eGFR) calculated through prediction equations derived from plasma or serum creatinine levels (8).

**Supplementary Table 1 . Comparison of Plasma Biomarkers Across CKD Stages with Adjustments for Age, Sex, and Amyloid Status**

| **Biomarker** | **Stage Comparison** | **p value (univariate)** | **p value (adjusted for age and sex)** | **p value (adjusted for age.sex and amyloid status)** |
| --- | --- | --- | --- | --- |
| Plasma Aβ42 | CKD stage1 - CKD stage2 | 0.021 | 0.019 | 0.010 |
| Plasma Aβ42 | CKD stage1 - CKD stage3 | 0.004 | 0.005 | 0.005 |
| Plasma Aβ42 | CKD stage2 - CKD stage3 | 0.053 | 0.062 | 0.076 |
| Plasma Aβ40 | CKD stage1 - CKD stage2 | <0.001 | 0.310 | 0.311 |
| Plasma Aβ40 | CKD stage1 - CKD stage3 | <0.001 | <0.001 | <0.001 |
| Plasma Aβ40 | CKD stage2 - CKD stage3 | 0.002 | 0.004 | 0.004 |
| Plasma Aβ42/40 | CKD stage1 - CKD stage2 | 0.763 | 0.095 | 0.064 |
| Plasma Aβ42/40 | CKD stage1 - CKD stage3 | 0.832 | 0.811 | 0.909 |
| Plasma Aβ42/40 | CKD stage2 - CKD stage3 | 0.934 | 0.997 | 0.940 |
| Plasma p-tau181 | CKD stage1 - CKD stage2 | 0.268 | 0.977 | 0.956 |
| Plasma p-tau181 | CKD stage1 - CKD stage3 | 0.033 | 0.126 | 0.054 |
| Plasma p-tau181 | CKD stage2 - CKD stage3 | 0.115 | 0.147 | 0.069 |
| Plasma p-tau217 | CKD stage1 - CKD stage2 | 0.585 | 0.838 | 0.685 |
| Plasma p-tau217 | CKD stage1 - CKD stage3 | 0.445 | 0.887 | 0.551 |
| Plasma p-tau217 | CKD stage2 - CKD stage3 | 0.662 | 0.785 | 0.380 |
| Plasma p-tau231 | CKD stage1 - CKD stage2 | 0.840 | 0.977 | 0.989 |
| Plasma p-tau231 | CKD stage1 - CKD stage3 | 0.192 | 0.394 | 0.253 |
| Plasma p-tau231 | CKD stage2 - CKD stage3 | 0.276 | 0.359 | 0.235 |
| Plasma NTA-tau | CKD stage1 - CKD stage2 | 1.000 | 0.986 | 0.976 |
| Plasma NTA-tau | CKD stage1 - CKD stage3 | 0.222 | 0.212 | 0.126 |
| Plasma NTA-tau | CKD stage2 - CKD stage3 | 0.237 | 0.236 | 0.148 |
| Plasma NfL | CKD stage1 - CKD stage2 | <0.001 | 0.289 | 0.300 |
| Plasma NfL | CKD stage1 - CKD stage3 | 0.000 | 0.000 | <0.001 |
| Plasma NfL | CKD stage2 - CKD stage3 | 0.000 | <0.001 | <0.001 |
| Plasma GfAP | CKD stage1 - CKD stage2 | <0.001 | 0.579 | 0.497 |
| Plasma GfAP | CKD stage1 - CKD stage3 | 0.162 | 0.628 | 0.346 |
| Plasma GfAP | CKD stage2 - CKD stage3 | 0.777 | 0.829 | 0.570 |

CKD, chronic kidney disease; Aβ42, amyloid-beta 42; Aβ40, amyloid-beta 40; p-tau181, phosphorylated tau at threonine 181; p-tau217, phosphorylated tau at threonine 217; p-tau231, phosphorylated tau at threonine 231; NTA = N-terminal containing tau fragments; NFL, neurofilament light chain; GFAP, glial fibrillary acidic protein; Aβ-PET, amyloid-beta positron emission tomography.

p-values from univariate and multivariate analyses comparing plasma biomarkers across different stages of CKD. The multivariate models are adjusted for age, sex, and amyloid status. Plasma biomarkers include Aβ42. Aβ40. Aβ42/40 ratio, p-tau181, p-tau217, p-tau231, NTA-tau, NfL, and GFAP.

**Supplementary Table 2. Comparison of Plasma Biomarkers Between Normal Renal Function and Mild Renal Impairment groups with Adjustments for Age, Sex, and Amyloid Status**

Aβ42, amyloid-beta 42; Aβ40, amyloid-beta 40; p-tau181, phosphorylated tau at threonine 181; p-tau217, phosphorylated tau at threonine 217; p-tau231, phosphorylated tau at threonine 231; NTA = N-terminal containing tau fragments; NFL, neurofilament light chain; GFAP, glial fibrillary acidic protein; Aβ-PET, amyloid-beta positron emission tomography.

p-values from univariate and multivariate analyses comparing plasma biomarkers between normal renal function and mild renal impairment groups. The multivariate models are adjusted for age, sex, and amyloid status. Plasma biomarkers include Aβ42. Aβ40. Aβ42/40 ratio, p-tau181, p-tau217, p-tau231, NTA-tau, NfL, and GFAP.

| Biomarker | Group Comparison | p value (univariate) | p value (adjusted for age and sex) | p value (adjusted for age,sex, and amyloid status) |
| --- | --- | --- | --- | --- |
| Plasma Aβ42 | Normal renal function - Mild renal impairment | 0,001 | 0,002 | 0,001 |
| Plasma Aβ40 | Normal renal function - Mild renal impairment | 0,002 | 0,032 | 0,033 |
| Plasma Aβ42/40 | Normal renal function - Mild renal impairment | 0,249 | 0,072 | 0,051 |
| Plasma p-tau181 | Normal renal function - Mild renal impairment | 0,116 | 0,504 | 0,442 |
| Plasma p-tau217 | Normal renal function - Mild renal impairment | 0,526 | 0,757 | 0,708 |
| Plasma p-tau231 | Normal renal function - Mild renal impairment | 0,442 | 0,897 | 0,845 |
| Plasma NTA-tau | Normal renal function - Mild renal impairment | 0,768 | 0,274 | 0,242 |
| Plasma NfL | Normal renal function - Mild renal impairment | 0,001 | 0,035 | 0,035 |
| Plasma GfAP | Normal renal function - Mild renal impairment | 0,013 | 0,260 | 0,186 |

**Supplementary table 3. Participant Characteristics and Plasma Biomarker Levels in Groups with Normal Renal Function and Mild Renal Impairment**

|  | **Normal Renal Function (N=147)** | **Mild Renal Impairment (N=95)** |
| --- | --- | --- |
| Age, median (Q1-Q3) | 67.1 (62.5-72.1) | 73.3 (69.4-77.9) |
| Female, n(%) | 97 (66.0%) | 63 (66.3%) |
| YOE, median (Q1-Q3) | 15.0 (12.0-17.0) | 15.0 (12.0-18.0) |
| MMSE, Median (Q1-Q3) | 29.0 (27.3-30.0)  [21] | 29.0 (26.0-30.0)  [8] |
| APOE ε4 carriers, No. (%) | 50 (34.0%)  [3] | 28 (29.5%)  [0] |
| Amyloid status, No. (%) | 61 (41.5%) | 39 (41.1%) |
| Plasma p-tau217, median (Q1-Q3), pg/mL | 0.305 (0.188-0.693)  [10] | 0.444 (0.276-0.847)  [13] |
| Plasma p-tau181, median (Q1-Q3), pg/mL | 7.98 (5.62-11.9)  [1] | 8.78 (6.95-13.0)  [1] |
| Plasma p-tau231, median (Q1-Q3), pg/mL | 14.1 (10.6-20.2)  [2] | 16.9 (12.8-22.6)  [1] |
| Plasma NTA-tau, median (Q1-Q3), pg/mL | 0.213 (0.118-0.410)  [4] | 0.258 (0.125-0.421)  [3] |
| Plasma GFAP, median (Q1-Q3), pg/mL | 149 (104-218)  [0] | 182 (148-255)  [1] |
| Plasma NfL, median (Q1-Q3), pg/mL | 18.8 (14.4-27.1)  [0] | 27.6 (18.7-33.6)  [1] |
| Plasma Aβ42, median (Q1-Q3), pg/mL | 6.17 (4.86-7.15)  [0] | 6.88 (5.90-7.89)  [1] |
| Plasma Aβ40, median (Q1-Q3), pg/mL | 89.6 (77.2-100)  [0] | 95.9 (85.4-109)  [1] |
| Plasma Aβ42/40, median (Q1-Q3), pg/mL | 0.0682 (0.0608-0.0789)  [0] | 0.0710 (0.0617-0.0830)  [1] |
|  |  |  |

Abbreviations: YOE = Years of Education; MMSE = Mini-Mental State Examination; APOE = Apolipoprotein E; eGFR = Estimated Glomerular Filtration Rate; p-tau = phosphorylated tau; NTA-tau = N-terminal containing tau fragments; GFAP = Glial Fibrillary Acidic Protein; NfL = Neurofilament Light chain; Aβ = Amyloid Beta.

Categorical variables are reported as counts and percentages, while continuous variables are presented as median values with interquartile ranges (Q1-Q3). Missing values are reported in brackets next to the respective variable

**Supplementary Figure 1. Associations between eGFR, age, and sex.**

A. Scatterplot indicating a significant association between age and eGFR, indicated by Spearman’s rho. B. Boxplots showing no differences between eGFR levels and sex, tested with a Wilcoxon signed-rank test. eGFR = estimated glomerular filtration rate.

**Supplementary Figure 2.** **Distribution of Alzheimer’s disease biomarkers across normal renal function and mild renal impairment categories**

Boxplots showing plasma levels of Alzheimer’s disease biomarkers (Aβ42, Aβ40, Aβ42/Aβ40, P-tau181, P-tau217, P-tau231, NTA-tau, NfL, and GFAP) in participants with normal renal function (eGFR > 90), mild renal impairment (eGFR 60-90), and eGFR < 60 (represented by different symbols). Participants are grouped into two categories: normal renal function and mild renal impairment. The majority of participants in the cohort fall within the normal to mild renal impairment range. Note the distribution of biomarker levels across the different renal function groups, as indicated by the varying symbols (circle for eGFR > 90, triangle for eGFR 60-90, and square for eGFR < 60).

**Supplementary Figure 3. Biomarker and eGFR levels in relation to risk of Aβ-positivity based on logistic regression models**

This plot represents the continuous and non-linear associations between biomarker and eGFR levels and the risk of Aβ-PET positivity, based on probabilities extracted from the full models containing plasma biomarker, eGFR, age and sex. Lines reaching values close to zero or one, or approaching a sigmoidal form, indicate the predictor in question is more strongly capable of discriminating between Aβ-negative and Aβ-positive individuals. Lines presenting more horizontally, as those seen for eGFR relation to risk of Aβ-PET positivity, indicate a low discriminatory and predictive value for the predictor.

**Supplementary Figure 4. Scatter Plots Depicting the Relationship Between eGFR and Plasma Biomarkers in Aβ-Positive and Aβ-Negative Individuals Using Log-Transformed Data**

**Supplementary Figure 4a – eGFR and BBM levels**

Scatter plots in the upper half of the layout showing the relationship between eGFR and Alzheimer's disease blood-based biomarkers (BBM) across the entire cohort using log-transformed data. The plots illustrate an inverse trend between eGFR and several biomarkers, including Aβ42, Aβ40, p-tau181, p-tau217, p-tau231, NfL, and GFAP. NTA-tau does not appear to show a significant relationship with eGFR. Grey points represent Aβ-negative individuals, while orange points represent Aβ-positive individuals. Non-linear regression lines, represented by solid curves, are fitted using a generalized additive model with cubic splines (3 knots), and the shaded areas around them indicate 95% confidence intervals. Spearman’s rho is used to numerically represent the associations.

**Supplementary Figure 4b – eGFR and BBM levels according to Aβ status**

Scatter plots illustrating the relationship between eGFR and Alzheimer's disease biomarkers, stratified by amyloid status (Aβ-negative and Aβ-positive). In the Aβ-negative group (grey points) using log-transformed data, an inverse relationship is seen between eGFR and several biomarkers, including Aβ42, Aβ40, p-tau181, p-tau217, p-tau231, NfL, and GFAP. In the Aβ-positive group (orange points), the relationship between eGFR and these biomarkers is generally weaker, with fewer noticeable trends compared to the Aβ-negative group. Non-linear regression lines, represented by solid curves, are fitted using a generalized additive model with cubic splines (3 knots), and the shaded areas around them indicate 95% confidence intervals. Spearman’s rho is used to numerically represent the associations.

**Supplementary Figure 5. Impact of eGFR on Alzheimer's Disease Biomarkers: Univariate and Multivariable Analyses based on Log-Transformed Data**

Forest plot displaying the standardized β-estimates of eGFR across various Alzheimer's Disease biomarkers, including Aβ42, Aβ40, Aβ42/Aβ40 ratio, P-tau217, P-tau231, NfL, and GFAP based on log-transformed data. The estimates are shown for three models: univariate (black), adjusted for age and sex (orange), and adjusted for age, sex, and Aβ-PET status (blue). Negative β-estimates indicate an inverse relationship between eGFR and biomarker levels.

**References**

1. Therriault J. Benedet AL. Pascoal TA. Mathotaarachchi S. Chamoun M. Savard M. et al. Association of apolipoprotein E ε4 with medial temporal tau independent of amyloid-β. JAMA neurology. 2020;77(4):470-9.

2. Hansen TI. Brezova V. Eikenes L. Håberg A. Vangberg TR. How does the accuracy of intracranial volume measurements affect normalized brain volumes? Sample size estimates based on 966 subjects from the HUNT MRI cohort. American Journal of Neuroradiology. 2015;36(8):1450-6.

3. Karikari TK. Pascoal TA. Ashton NJ. Janelidze S. Benedet AL. Rodriguez JL. et al. Blood phosphorylated tau 181 as a biomarker for Alzheimer's disease: a diagnostic performance and prediction modelling study using data from four prospective cohorts. The Lancet Neurology. 2020;19(5):422-33.

4. Ashton NJ. Pascoal TA. Karikari TK. Benedet AL. Lantero-Rodriguez J. Brinkmalm G. et al. Plasma p-tau231: a new biomarker for incipient Alzheimer’s disease pathology. Acta neuropathologica. 2021;141:709-24.

5. Lantero-Rodriguez J. Salvadó G. Snellman A. Montoliu-Gaya L. Brum WS. Benedet AL. et al. Plasma N-terminal containing tau fragments (NTA-tau): a biomarker of tau deposition in Alzheimer’s Disease. Molecular Neurodegeneration. 2024;19(1):19.

6. Ashton NJ. Brum WS. Di Molfetta G. Benedet AL. Arslan B. Jonaitis E. et al. Diagnostic accuracy of a plasma phosphorylated tau 217 immunoassay for Alzheimer disease pathology. JAMA neurology. 2024;81(3):255-63.

7. Inker LA. Eneanya ND. Coresh J. Tighiouart H. Wang D. Sang Y. et al. New creatinine-and cystatin C–based equations to estimate GFR without race. New England Journal of Medicine. 2021;385(19):1737-49.

8. Foundation NK. KDOQI clinical practice guidelines. Available from: <https://www.kidney.org/professionals/kdoqi>. Accessed September 30, 2024.
